# The riddle of shiftwork and disturbed chronobiology: a case study of landmark smoking data demonstrates fallacies of not considering the ubiquity of an exposure

**DOI:** 10.1186/s12995-020-00263-2

**Published:** 2020-05-19

**Authors:** Thomas C. Erren, Philip Lewis, Peter Morfeld

**Affiliations:** Institute and Policlinic for Occupational Medicine, Environmental Medicine and Prevention Research, 50937 Cologne, Germany

**Keywords:** Bias, Exposure assessment, Information bias, Circadian, Chronobiology, Night work, Shiftwork, Cancer

## Abstract

**Background:**

Failing to integrate all sources of a ubiquitous hazard candidate may explain inconsistent and/or null, and overall misleading, results in epidemiological studies such as those related to shift-work.

**Methods:**

We explore this rationale on the assumption that Doll and Hill had confined their 1950 landmark study to smoking at workplaces alone. We assess how non-differential, or how differential, underestimation of exposure could have biased computed risks.

**Results:**

Systematically unappreciated exposures at play could have led to substantial information bias. Beyond affecting the magnitude of risks, not even the direction of risk distortion would have been predictable.

**Conclusions:**

Disturbed chronobiology research should consider cumulative doses from all walks of life. This is a *conditiosine qua non *to avoid potentially biased and uninterpretable risk estimates when assessing effects of a ubiquitous hazard candidate.

## Introduction

Working and living against inner clocks is bad for health, or so it goes. As one example, most of us recognise the effects of jet lag as – albeit short-term – consequences of time-zone travel and disturbed chronobiology [1]. We might then expect medium- and long-term shiftwork against inner clocks to affect humans more chronically, plausibly being more detrimental to individual and population health. Clearly, an abundance of laboratory-based evidence and chronobiological rationale suggest that this is true [2–5]. Surprisingly then, despite numerous studies into various adverse health effects, no recognized occupational disease is associated with working against endogenous clocks, except for Denmark where breast cancer related to night work may be recognized on a case-by-case basis [6]. So far, epidemiology fails to observe associations which we expect in a consistent fashion. One example is that epidemiology studies yield inconsistent and, overall, limited evidence of links between shiftwork and cancer [7, 8]. The riddle – and outstanding question – is ‘why’? [9].

Before we accept the epistemologically conceivable null hypothesis – i.e. that working against inner clocks and disturbed chronobiology does not significantly impair health – we should step back and look at how disturbed chronobiology and plausibly associated effects are researched in the first place. Unavoidably, dose considerations come into play. Do we validly capture doses of disturbed chronobiology in different working populations? With regard to exposure gradients in traditional shiftwork epidemiology, we suspect not [10, 11]. In our view, assessing doses of assumed disturbed chronobiology via shiftwork information alone may underestimate true exposures from both work and play. Further epidemiological pitfalls may include the ‘healthy worker effect’ (HWE) [12, 13] wherein workers who feel stressed, strained, and generally unwell may reduce work-associated causes of disturbed chronobiology before manifest disease [14]. This can lead to selection-out effects and information bias in epidemiological studies in occupational settings [15].

In this paper, we quantitatively explore our hypothesis that cumulative doses must combine ubiquitous sources of disturbed chronobiology [10–14, 16] via an analogy with smoking research:

### Analogy

1. Both smoking and disturbed chronobiology expose individuals both at work and at play.
2. What could be associated risks if smoking – like disturbed chronobiology – had been targeted only at work?

Insofar, we expect both smoking and disturbed chronobiology to be a risk exposure at work *and* play. Next, we explore possible effects on “empirical” risks had Doll & Hill confined their assessment of smoking – analogous with how shiftwork epidemiology targets disturbed chronobiology – to workplaces alone. Specifically, we quantify what may happen when only partial doses of a ubiquitous culprit are considered by using the 1950 data into smoking and lung cancer risks. A discussion of the implications of information bias in traditional shiftwork epidemiology and a plea to consider doses of disturbed chronobiology both at and off work close this paper.

## Methods

We use data provided by the seminal Doll & Hill (1950) paper [17] to illustrate hypothetical information bias on determining risk estimates in smoking research. Specifically, respective data on the most recent regular amount of cigarettes smoked per day before the onset of illness in *n* = 647 male lung cancer patients and *n* = 622 control patients were utilised to develop six scenarios of exposure underestimation. Firstly, and for simplicity later, we used the cut-points of the five dose categories in Doll & Hill’s Table V (those who smoked 1-, 5-, 15-, 25-, or 50+ cigarettes per day; pg.742) [17] to become Table 1 in our paper (those who smoked 1, 5, 15, 25, or 50 cigarettes per day). Then, we collapsed the five dose categories into two categories (those who smoked 20 cigarettes or less per day and those who smoked more than 20). As example, if an individual smoked, for instance, 50 cigarettes per day, he/she would be included in the > 20 per day group. Assuming all cigarettes smoked are relevant to the outcome, this distribution is our scenario 1 (S1).

**Table 1:** Modified from Doll & Hill 1950 (Table V, p. 742) [17]

“Tabel V. – Most recent [number of ciggarettes] consumed regularly by smokers before the onset of present illness”					
Disease Group	No. Smoking Daily^**a**^				
	1 Cig.	5 Cigs.	15 Cigs.	25 Cigs.	50 Cigs.
**Males:** **Lung-carcinoma patients (647)**	33	250	196	136	32
**Control patients with diseases other than cancer (622)**	55	293	190	71	13

^a^For simplicty of calculations, we used the cut-points of Doll’s & Hill’s original table to be the total amount of cigarrettes smoked by these individuals

**Fig. 1:**
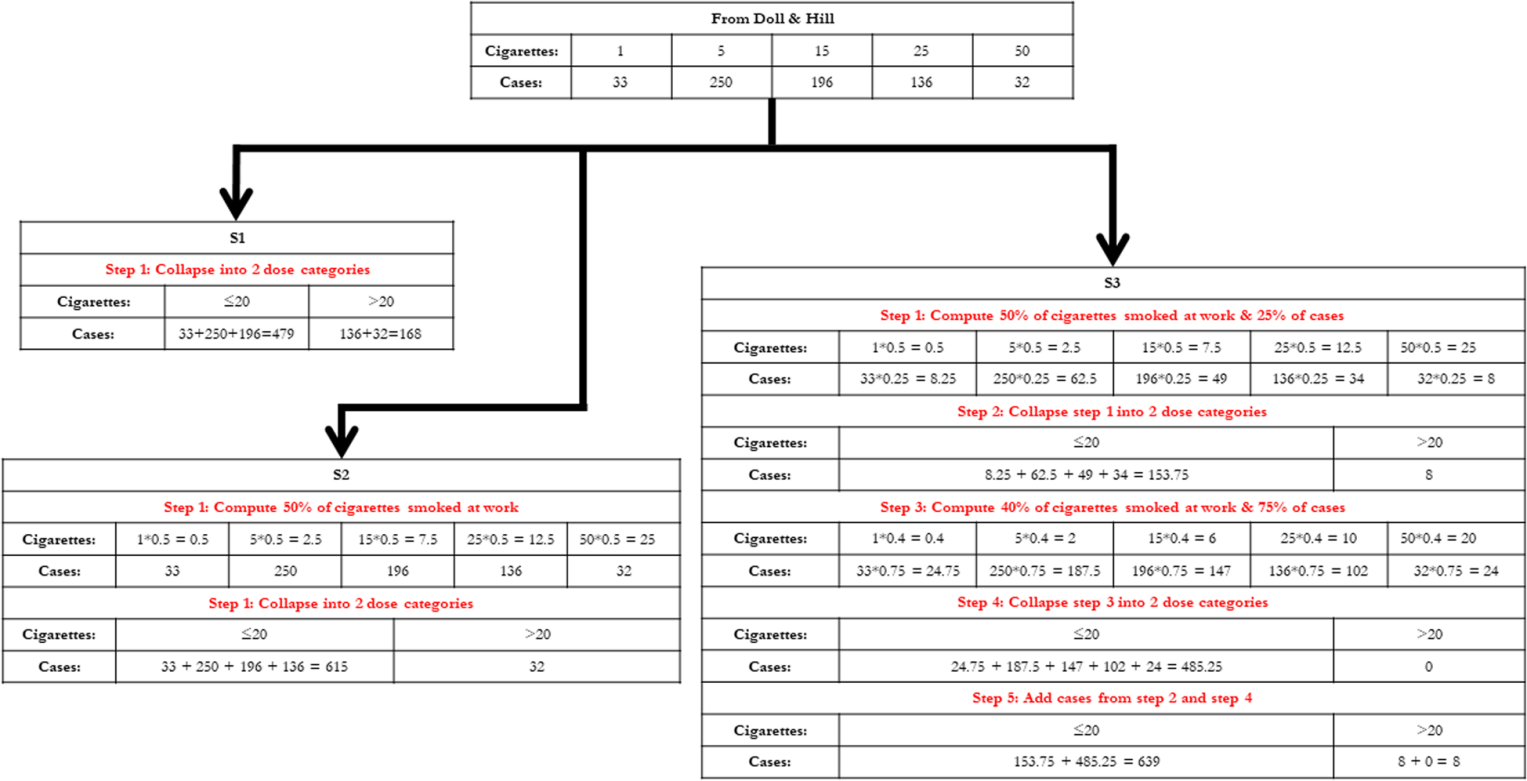
Step-by-step to computing number of cases in S1, S2, and S3. Number of controls in S1, S2, and S3 are calculated accordingly. To derive S4, S5, and S6, simply change the dose categories from 20 and > 20 to 10 and > 10 for each step and modify the number of cases/controls at each step accordingly

For scenario 2 (S2) and scenario 3 (S3), only cigarettes smoked at work are hypothesized to be relevant to the outcome. S2 is a scenario with non-differential exposure underestimation. For S2, number of cigarettes smoked per day was assumed evenly distributed across times of work and times of play, irrespective of being a case or control. To exemplify, if an individual smoked, for instance, 50 cigarettes per day, he/she would now be considered to have smoked only 25 cigarettes at work. S3 is the same as S2 except the number of cigarettes smoked per day was assumed unevenly distributed between times of work and times of play among cases and for each of the 5 dose categories in Table 1 (75% of cases in each category smoked 40% at the work place and 25% of cases smoked 50% at the workplace). If the above example of a smoker of 50 cigarettes belonged to the 75% or 25% of cases, he/she would now be considered as having smoked 20 or 25 cigarettes at work, respectively. Cases were redistributed in the two dose categories accordingly. Controls are as in S2. Thus, S3 is a scenario with differential exposure underestimation; that is to say, the exposure underestimation differs between cases and controls. The derivation of case numbers in S1-S3 is illustrated in Fig. 1.

We developed a further three scenarios with a different dose cut-off point. Scenarios 4, 5, and 6 (S4, S5, and S6) are the same as S1-S3 respectively, except the two dose categories comprised those who smoked 10 cigarettes or less per day and those who smoked more than 10. S5 is based on non-differential exposure underestimation whereas S6 is based on differential exposure underestimation.

Odds ratios (ORs) and 95% confidence intervals (CIs) were computed for each scenario. We combined S2 and S1 tables and fitted a logistic regression model with two binary baseline terms (cigarette dose, table indicator) and one interaction term (product term: cigarette dose * table indicator). The interaction term measures how much the OR in scenario S2 differs from the OR in S1, i.e., it measures direction and magnitude of the bias and can be represented by an OR with 95% CIs. These 95% CIs were calculated under the (wrong) assumption that the deviations between Tables S2 and S1 were random only so that the CIs can be used to evaluate whether the bias is pronounced compared with random uncertainties of the study. We also contrasted S3 and S1, S5 and S4, and S6 and S4. Calculations were made with Stata, Version 14 (StataCorp, LLC, College Station, TX, USA).

## Results

The distribution of cases and controls in the two dose categories varied by scenario (Table 2). In S2, where an even distribution of smoking across work and play was assumed and only smoking at work was hypothesized as biologically relevant, there are much fewer individuals in the more than 20 cigarettes per day category and vice versa for the 20 cigarettes or less per day category. In S3, the 25% of cases smoking 50% at work means only 25% of S2 cases are counted in the more than 20 cigarettes per day category. As the other 75% cases were assumed to have smoked less than 50% at the work place, they are categorized as having smoked less than 20 cigarettes per day. The distribution of cases and controls between the dose categories in S4 is much more even compared to S1 while the distribution for S6 is less even compared to S3.

**Table 2:** Cancer cases and controls per dose category and scenario with associated lung cancer ORs and corresponding 95% CIs

		Most recent regular amount of cigarettes smoked per day before the onset of illness			
		≤ 20	> 20	OR	95% CI
**S1**	**Controls**	538	84		
	**Cases**	479	168	2.25	1.67–3.04
**S2**	**Controls**	609	13		
	**Cases**	615	32	2.44	1.23–5.11
**S3**	**Controls**	609	13		
	**Cases**	639	8	0.59	0.21–1.54
		**≤ 10**	**> 10**		
**S4**	**Controls**	348	274		
	**Cases**	283	364	1.63	1.3–2.05
**S5**	**Controls**	538	84		
	**Cases**	479	168	2.25	1.67–3.04
**S6**	**Controls**	538	84		
	**Cases**	581	66	0.73	0.51–1.04

S1 & S4: cumulative smoking at work + at play

S2 & S5: cumulative smoking at work assuming even distribution between at work + at play

S3 & S6: cumulative smoking at work assuming uneven distribution between at work + at play for cases (75% cases in each Table 1 category with 40% of smoking at work; 25% cases in each Table 1 category with 50% at work) and an even distribution for controls

Computed ORs and 95% CIs for S1-S6 are also presented in Table 2. In S1, taking cigarettes smoked at work and at play into account, individuals who smoked more than 20 cigarettes per day presented with OR 2.25 (95% CI 1.67–3.03) compared to those who smoked 20 or less per day. In S2, with lung cancer risks now – erroneously – attributed to smoking at work alone, the OR rises to 2.44 (95% CI 1.23–5.01). S3 leads to an underestimation of the effects of smoking on lung cancer, with OR decreasing to 0.59 (95% CI 0.21–1.54). Similar result patterns for S4-S6 are observed although quantitatively different.

The OR underestimation in S3 is very pronounced compared to S1 and S2, evinced by the fact that the size of the bias is not covered by the confidence intervals. The intervals of the S1 and S3 results do not overlap and the OR for interaction is 0.26 (95% CI 0.10– 0.66). The OR overestimation in S2, on the other hand, is weak as evinced by comparing the width of the confidence intervals in S1 and S2. The confidence interval for S1 lies completely within the confidence interval for S2. The OR for interaction is only 1.09 (95% CI 0.53–2.22).

The OR for S5 vs. S4 interaction is 1.38 (95% CI 0.95–1.98). The overestimation is therefore more pronounced than in the example of S2 vs. S1 and the lower limit is close to 1. The OR for S6 vs. S4 interaction is 0.45 (95% CI 0.30–0.67); thus, similar to S3 vs. S1, the estimated bias is very pronounced.

## Discussion

Had landmark 1950s research assessed smoking at workplaces alone, both non-differential *and* differential exposure underestimation could have led to substantially biased risk estimates. Indeed, beyond affecting the magnitude of resulting lung cancer risks, not even the direction of risk distortion could have been predicted. It follows that a hazard suspected to be detrimental at work and play – be it smoking or be it disturbed chronobiology – must be fully assessed and appreciated for meaningful interpretations.

Information bias in the form of non-differential misclassification of exposure is typically considered to lead to an underestimate of the exposure-disease association [15]. This is because exposures of cases are underestimated but those of controls are overestimated (given a true OR > 1). We note that the information bias in S2 and S5 is due to a non-differential but systematic underestimation of exposures in cases and controls. This led to risk overestimation compared to S1 or S4. Thus, the biases we describe here should not be confused with usual effects of non-differential misclassification [15]. Furthermore, the question arises whether potential overestimations are always relatively unimportant, i.e. that they cannot add significantly to the uncertainty stemming from the random errors described by the confidence intervals (S2 vs. S1: OR = 1.09, 95% CI 0.53–2.22). In the case of S5 vs. S4 the bias is larger (OR = 1.38) and more pronounced (95% CI 0.95–1.98).

The downward bias in S3 and S6 (scenarios of differential underestimation of exposure) may be explained by a typical HWE [12, 13] as workers who become ill or particularly stressed and strained may reduce work place exposures before manifest illness [14] A second aspect, which must be considered, is that the study by Doll & Hill is cross-sectional in design with regard to the exposure. As smoking is not recorded in a longitudinal fashion, only the prevalence of exposure is determined at a relatively late point in time before diagnosis of disease. HWE effects are known to have a strong effect in cross-sectional studies [18]. If the exposure is measured in a longitudinal fashion, this reduces the influence of a HWE, but does not eliminate it. HWE effects also occur in longitudinal studies [15, 19–21].

The assumptions made for S3 and S6 appear realistic when based on the description of Doll’s & Hill’s Table V, according to which the amount of smoked cigarettes was determined shortly before the onset of the disease. Note that “In Table V they [=smokers] have been subdivided according to the amount they smoked immediately before the onset of the illness which brought them into hospital. (If they had given up smoking before then, then they have been classified according to the amount smoked immediately prior to giving it up.)” On that information basis, it appears plausible that 75% of soon-to-be-diagnosed cases with significant precursors or already manifest cancer reduced their smoking at work but that unaffected controls did not. In effect, physically challenged workers may have reduced smoking where, and when, they had to cope with extensive cardio-pulmonary strain due to work-associated manual labour. In this scenario, it is also possible that smoking-addicted cases could have compensated at play for their moderately reduced smoking at work. Note that, while the specific information in Table V (“Most Recent Amount of Tobacco* Consumed Regularly”) allows to set up S3 and S6, information on cumulative lifetime cigarette exposure and longitudinal dose information would probably not.

Importantly, the simplification made at the beginning (i.e. using cut-points of intervals) is not the ‘origin’ of the information bias shown. In principle, similar scenarios can be derived if we were to assume, for example, uniformly distributed smoking intensities within the categories of Table 1. However, the resulting Table 2 would be more difficult to grasp. Thus, we offer the simplification purely for general illustration. Of course, assuming uniformly distributed smoking categories will result in some loss of resolution but the points made still stand.

Our illustrations can be seen as a concrete example of measurement error scenarios studied in detail more than two decades ago. Brenner (1993) and Brenner & Loomis (1994) investigated the direction and magnitude of bias in relative risk estimates due to differential and non-differential exposure measurement errors [22, 23]. They noted that “exposure to environmental hazards is often underestimated if the study focuses on only one of several possible sources (for example, occupational exposure)” [23]. Brenner & Loomis concluded from their analyses: “Systematic non-differential over- or underestimation of the exposure may bias measures of the exposure-disease association either toward the null or away from the ‘null’ and emphasized that violations of the non-differentiality assumption may lead to strong biases in any direction [23]. The authors added: “If exposure measurement error has both random and systematic components, the direction of the net bias is less predictable than with pure error of either type” [23]. All this is in line with our results: If disturbed chronobiology is a ubiquitous hazard, research to-date that has targeted exposures at work but not at play may be uninterpretable due to unpredictable biases.

Taken together, based on “analogously” truncating exposure at play in smoking research, our demonstrations imply that – if disturbed chronobiology at work *and* at play were detrimental to humans – results in studies with lifelong shiftwork exposure can be distorted if we restrict observations regarding disturbed chronobiology to working hours. In other words: An information bias of the kind put forward by Rothman et al. 2008 will occur [15]. That this information bias can erroneously produce effects in both directions, cannot be stated too strongly: upwards for non-differential underestimation of exposure (S2 and S5) or downwards for differential underestimation of exposure in the sense of a HWE (S3 and S6).

In principle, disturbed chronobiology at play may be significant and relevant. For instance, sleep timing can be different between a 5-day working week and a 2-day weekend for many fixed day workers [24] who would be classified as not exposed to disturbed chronobiology in traditional night (shift) work epidemiology. Even if this difference is a few hours, the push or pull on phase of circadian rhythms may result in transient periods of circadian misalignment and, thus, only a few days per week of circadian alignment. This could amount to years of misalignment over a person’s working life [25]. This may be taken further as sleep deficiency is a problem more generally [26, 27]. Could disturbed chronobiology from time at play equate to disturbed chronobiology from time at work in fixed day workers? Empirically, the answer to this question is open. In the outlined group, however, we would expect more disturbed chronobiology from time at play than from time at work. Disturbed chronobiology for a shift-working group will depend on determinants such as chronotype (an individual’s preferential timing for being awake and asleep), shift-schedule, and lifestyle habits (for instance, individuals’ social behaviour over weekends or other free days in conflict with their chronotype) more generally. How much disturbed chronobiology may be accumulated at work and/or at play appears – at this stage of very limited empirical insights into determinants of disturbed chronobiology in either setting – unpredictable. Disconcertingly, effects of the information bias which we quantified via an analogy with smoking research may apply to what occurs in shift-work-confined epidemiological studies of disturbed chronobiology.

## Conclusion

We conclude that non-consideration, or truncation, of relevant doses due to information bias can lead to pronounced errors. Moreover, the hypothesis that disturbed chronobiology at work *and* at play is detrimental to humans is not falsified. To the contrary, regarding possible links between disturbed chronobiology and cancer, experts convened by the International Agency for Research on Cancer [IARC] concluded in 2007: There is “sufficient evidence in experimental animals for the carcinogenicity of light during the daily dark period (biological night)” [7]. In 2019, a further IARC Working Group concluded that evidence of cancer is sufficient, and mechanistic evidence strong, when animals are exposed to alterations in the light–dark schedule which shiftworkers experience [8]. In both instances of evaluation, “limited evidence of cancer in humans” was the weakest stream of evidence when evaluating carcinogenicity. The obvious question is “why?”

Epistemologically, one answer could be that epidemiology to-date considered only partial doses when assessing effects of a ubiquitous hazard candidate. Whenever our bodies don’t expect it, exposures to light and/or work/activities [28] and/or eating [29] and/or noise can cause disturbed chronobiology, be that at work and/or at play. Thus, why should causes and effects of disturbed chronobiology be limited to exposures at the workplace?

To answer this question, to contribute to solving the riddle surrounding shift-work studies, and to possibly identify a ubiquitous hazard, it is a *conditiosine qua non *to avoid the demonstrated dose fallacies.

## Data Availability

All data required to replicate this study is available in the manuscript.

## Abbreviations

HWE: Healthy Worker Effect
S1–6: Scenarios 1–6
OR: Odds Ratio
CIs: Confidence Intervals
IARC: International Agency for Research on Cancer
